# Verification of the Short-Term Impact of Imeglimin on Liver Fibrosis Markers Stratified by Liver Fibrosis Risk in Patients With Type 2 Diabetes

**DOI:** 10.7759/cureus.82625

**Published:** 2025-04-20

**Authors:** Takumi Tanaka, Takashi Kitao, Motohiro Kubori, Yoshio Komoda, Yukiko Mori, Takeshi Ibata

**Affiliations:** 1 Department of Diabetes, Endocrinology and Metabolism, Minoh City Hospital, Minoh City, JPN; 2 Department of Cardiology, Minoh City Hospital, Minoh City, JPN

**Keywords:** apri, fib-4 index, imeglimin, masld, type 2 diabetes

## Abstract

Objective

This study aimed to evaluate the effects of imeglimin on Fibrosis-4 index (FIB-4) and aspartate aminotransferase to platelet ratio index (APRI) across three subgroups classified according to the FIB-4 classification for assessing liver fibrosis risk in patients with type 2 diabetes mellitus (T2DM).

Materials and methods

Eighty-three patients with T2DM were classified into three subgroups based on their FIB-4 at the initiation of imeglimin, following the FIB-4 classification: Group 1 (G1) (FIB-4 < 1.30, n = 25), Group 2 (G2) (1.30 ≤ FIB-4 < 2.67, n = 44), and Group 3 (G3) (FIB-4 ≥ 2.67, n = 14). Then we evaluated the changes (Δ) in FIB-4 and APRI three months after the initiation of imeglimin in each subgroup. Subsequently, ΔFIB-4 and ΔAPRI were compared across the three subgroups. Baseline parameters and their changes correlated with ΔFIB-4 were also analyzed.

Results

FIB-4 significantly decreased in G2 (p = 0.046) and G3 (p = 0.017), while APRI showed significant reductions across all three subgroups (G1: p = 0.007, G2: p < 0.001, G3: p = 0.002). ΔFIB-4 in G3 was significantly greater than that observed in G1 (p = 0.01), and ΔAPRI in G3 was significantly greater than those in G1 (p = 0.004) and G2 (p = 0.007). ΔFIB-4 was negatively correlated with baseline FIB-4 and Triglycerides (TG), and positively correlated with Δγ-glutamyl transpeptidase (γ-GTP).

Conclusions

The short-term effects of imeglimin on FIB-4 and APRI in patients with T2DM may be more pronounced in patients with higher baseline FIB-4 levels.

## Introduction

Hepatic steatosis in individuals with type 2 diabetes mellitus

In 2023, the nomenclature for non-alcoholic fatty liver disease (NAFLD) and non-alcoholic steatohepatitis (NASH) was revised to metabolic dysfunction-associated steatotic liver disease (MASLD) and metabolic dysfunction-associated steatohepatitis (MASH), respectively, accompanied by partial revisions to the diagnostic criteria [1]. The diagnosis of MASLD requires the presence of metabolic dysfunction, thereby placing greater emphasis on its association with metabolic abnormalities compared to the previous definition of NAFLD. In this article, the terms MASLD and MASH are used throughout in place of NAFLD and NASH, respectively, even when referring to studies conducted prior to the change in nomenclature. 

Approximately 70% of individuals with type 2 diabetes exhibit fatty liver [2], and MASLD has been shown to more than double the risk of developing type 2 diabetes [3], underscoring a strong interrelationship between the two conditions. Furthermore, the concurrence of diabetes and MASLD serves as a significant risk factor for the advancement of liver fibrosis [4], while also elevating the likelihood of chronic kidney disease (CKD) [5], liver-related complications, and overall mortality [6]. Individuals with both diabetes and MASLD exhibit a twofold increased risk of cardiovascular disease (CVD) compared to individuals with diabetes alone [7], indicating the compounding effect of MASLD on CVD risk among patients with type 2 diabetes. Despite being the gold standard for fibrosis assessment, liver biopsy’s invasiveness and procedural risks have driven the adoption of noninvasive tests (NITs) as essential tools for diagnosing, monitoring, and prognosticating MASLD [8].

Prognostic and predictive utility of liver fibrosis markers

Liver fibrosis is recognized as a critical determinant of liver-related mortality and overall prognosis [9], and although previously considered irreversible, recent studies have reported that liver fibrosis is reversible, even at an advanced stage [10]. Noninvasive assessment methods for liver fibrosis include the NAFLD fibrosis score (NFS) [11], the Fibrosis-4 index (FIB-4) [12], and the aspartate aminotransferase-to-platelet ratio index (APRI) [13]. FIB-4 has been identified as a predictive marker for ischemic heart disease in individuals with fatty liver [14]. The FIB-4, NFS, and APRI scores are correlated with a higher prevalence of heart failure [15], establishing liver fibrosis markers as prognostic indicators not only for liver disease but also for cardiovascular disease.

Hepatic effects of imeglimin

Imeglimin shares structural similarities with metformin and, in addition to enhancing insulin sensitivity like metformin, also promotes insulin secretion in a glucose concentration-dependent manner. Evidence suggests that imeglimin exerts its effects on the pancreas, skeletal muscle, and liver by targeting mitochondria and mitigating oxidative stress. Imeglimin ameliorates mitochondrial dysfunction in the liver and enhances hepatic insulin sensitivity [16]. The hepatoprotective effects of imeglimin are reportedly mediated, at least in part, through its action on mitochondria. A recent study indicates that imeglimin significantly ameliorates liver inflammation and fibrosis in patients with type 2 diabetes and MASLD [17]. However, limited data exist regarding its hepatic effects, particularly on liver fibrosis markers across varying severities of fibrosis. This study investigates the hepatic effects of imeglimin in diabetic patients stratified by the FIB-4, a key marker of liver fibrosis.

## Materials and methods

Study design and subjects

This single-center, retrospective, observational study utilized a prospectively maintained database. A total of 175 consecutive patients with type 2 diabetes mellitus (T2DM) who initiated imeglimin between September 2022 and May 2024 were initially identified. Of these, patients were excluded if they were under 20 years old (one patient), had initiated antidiabetic, antihypertensive, or lipid-lowering medications within three months before or after imeglimin initiation (35 patients), were not followed up at 3 months after imeglimin initiation (42 patients), discontinued imeglimin within three months (10 patients), or had incomplete data (four patients). After applying these exclusion criteria, 83 patients were included in the final analysis. The study adhered to the ethical principles of the Declaration of Helsinki, and the protocol was approved by the Minoh City Hospital Ethics Committee (No. R0610B50). Informed consent was obtained via an opt-out procedure.

Imeglimin dosage

Imeglimin was predominantly initiated at a dosage of 2,000 mg/day, with some patients receiving an initial dose of 1,000 mg/day at the discretion of their physician.

Data collection

We extracted demographic information, including age, sex, body mass index, as well as clinical data such as medical history, comorbidities, medications, and laboratory data from medical records. Baseline laboratory data were evaluated at the initiation of imeglimin. AST and ALT levels were measured at our institutional central laboratory using the IFCC (International Federation of Clinical Chemistry and Laboratory Medicine) method, which was consistently applied throughout the study period. Patients were monitored at least monthly by the attending physician, with blood tests at each visit. FIB-4 and APRI values at baseline and three months after the initiation of imeglimin were analyzed.

Classification of subjects

Eighty-three patients with T2DM were classified into three subgroups based on their FIB-4 at the initiation of imeglimin in accordance with the FIB-4 classifications, which was a tool for assessing liver fibrosis risk in patients with MASLD, as outlined in the evidence-based clinical practice guidelines for nonalcoholic fatty liver disease/nonalcoholic steatohepatitis 2020 [18]: Group 1 (G1) (low risk, FIB-4 < 1.30, n = 25), Group 2 (G2) (middle risk, 1.30 ≤ FIB-4 < 2.67, n = 44), and Group 3 (G3) (high risk, FIB-4 ≥ 2.67, n = 14).

Outcomes

We evaluated the changes (Δ) in FIB-4 and APRI three months after the initiation of imeglimin in each subgroup. Subsequently, ΔFIB-4 and ΔAPRI were compared across the three subgroups. Baseline parameters and their changes correlated with ΔFIB-4 were also analyzed.

Statistical analysis

Continuous variables are presented as medians (interquartile range), while categorical data are expressed as percentages. Statistical significance was assessed using the Wilcoxon signed-rank test, the Kruskal-Wallis test, and the Steel-Dwass test for continuous variables, and Fisher’s exact test for categorical variables [19]. Multiple regression analysis was performed to assess the impact of baseline parameters and their changes on ΔFIB-4, with all parameters standardized before analysis. Independent variables were initially selected based on their clinical relevance. The variance inflation factor (VIF) was calculated to assess multicollinearity. In cases where a high VIF was observed, the pair of correlated variables was examined, and the more clinically significant variable was retained. As a result, the following variables were included in the regression models: baseline values of FIB-4, hemoglobin A1c (HbA1c), γ-glutamyl transpeptidase (γ-GTP), and triglyceride (TG) for the first model, and changes in γ-GTP, HbA1c, TG, and low-density lipoprotein cholesterol (LDL-C) over the study period for the second model. A significance level of P < 0.05 was considered statistically significant. All statistical analyses were conducted using Python (version 3.11.11) with scikit-posthocs (version 0.11.1), scipy (version 1.13.1), statsmodels (version 0.14.4).

## Results

Patient characteristics at the initiation of imeglimin across the three subgroups

Table 1 presents the patient characteristics across the three subgroups, classified based on their FIB-4 at the initiation of imeglimin, in accordance with the FIB-4 classification for assessing liver fibrosis risk.

**Table 1:** Baseline characteristics of patients at the initiation of imeglimin across three subgroups, classified based on their FIB-4 index Continuous variables are presented as median (interquartile range). Categorical variables are presented as n (%). Tests for significance were conducted using the Kruskal-Wallis test for continuous variables and Fisher’s exact test for categorical data. Asterisks (*) indicate statistically significant p-values. Abbreviations: ACEi, angiotensin-converting enzyme inhibitor; ALT, alanine transaminase; APRI, aspartate aminotransferase to platelet ratio index; ARB, angiotensin II receptor blocker; AST, aspartate aminotransferase; DPP-4i, dipeptidyl peptidase-4 inhibitor; eGFR, estimated glomerular filtration rate; FIB-4 index, fibrosis-4 index; GLP-1RA, glucagon-like peptide-1 receptor agonist; γ-GTP, γ-glutamyl transpeptidase; HbA1c, hemoglobin A1c; HDL-C, high-density lipoprotein cholesterol; LDL-C, low-density lipoprotein cholesterol; MRA, mineralocorticoid receptor antagonist; SGLT2i, sodium-glucose cotransporter 2 inhibitor; TG, Triglyceride

Clinical data	Overall (n = 83)	FIB-4 index			p-value
		< 1.30 (G1, n = 25)	1.30 ≤, < 2.67 (G2, n = 44)	2.67 ≤ (G3, n = 14)	
FIB-4 index	1.68 (1.18, 2.40)	0.96 (0.9, 1.12)	1.81 (1.63, 2.18)	3.06 (2.81, 3.39)	ー
APRI	0.29 (0.23, 0.39)	0.20 (0.16, 0.23)	0.29 (0.25, 0.37)	0.58 (0.42, 0.67)	< 0.001*
Imeglimin dosage (mg/day)	2000 (1000, 2000)	2000 (2000, 2000)	2000 (1000, 2000)	2000 (1000, 2000)	0.38
Age (years)	70 (61, 76)	59 (47, 65)	73 (68, 78)	76 (60, 81)	< 0.001*
Male	45 (54%)	12 (48%)	24 (55%)	9 (64%)	0.62
Body mass index (kg/m^2^)	23.8 (21.7, 25.2)	25.1 (24.1, 27.0)	22.5 (20.9, 24.3)	24.1 (21.7, 26.3)	<0.001*
Systolic blood pressure (mmHg)	127 (121, 137)	127 (121, 137)	126 (120, 140)	133 (126, 137)	0.27
Heart rate (bpm)	80.0 (71.0, 89.0)	72.0 (70.0, 80.0)	83.0 (72.0, 88.0)	89.0 (79.5, 92.0)	0.046*
Duration of diabetes (years)	13 (9, 21)	13 (9, 20)	16 (11, 21)	13 (6, 19)	0.73
Hypertension	58 (70%)	16 (64%)	30 (68%)	12 (86%)	0.34
Dyslipidemia	66 (80%)	21 (84%)	36 (82%)	9 (64%)	0.29
Chronic kidney disease	26 (31%)	4 (16%)	17 (39%)	5 (36%)	0.14
Current Smoker	10 (12%)	4 (16%)	4 ( 9%)	2 (14%)	0.67
Laboratory data					
Hemoglobin (g/dL)	14.1 (13.2, 15.1)	14.7 (14.1, 15.9)	13.7 (12.8, 14.2)	14.2 (13.4, 15.7)	0.009*
Platelet (×10^ 4^ /μL)	20.2 (16.8, 23.9)	24.9 (21.7, 26.9)	19.4 (17.2, 21.5)	14.4 (12.9, 16.3)	< 0.001*
HbA1c (%)	8.4 (7.8, 9.8)	8.7 (7.8, 10.7)	8.45 (7.9, 9.4)	8.1 (7.7, 9.6)	0.76
eGFR (mL/min/1.73 m^2^)	69 (58, 88)	84 (65, 97)	62 (56, 72)	75 (53, 94)	0.003*
serum Albumin (g/dL)	4.2 (4.0, 4.4)	4.3 (4.2, 4.5)	4.2 (4.0, 4.4)	4.1 (3.9, 4.3)	0.08
Uric acid (mg/dL)	5.0 (4.0, 5.4)	5.1 (4.3, 5.6)	4.8 (3.7, 5.4)	4.8 (3.6, 5.2)	0.46
AST (IU/L)	22 (18, 27)	19 (16, 23)	22 (19, 26)	32 (26, 50)	< 0.001*
ALT (IU/L)	21 (16, 30)	22 (18, 30)	20 (15, 26)	29 (18, 34)	0.14
γ-GTP (IU/L)	28 (19, 48)	31 (21, 46)	25 (18, 39)	53 (26, 120)	0.033*
Total bilirubin (mg/dL)	0.60 (0.47, 0.84)	0.62 (0.46, 0.96)	0.55 (0.48, 0.80)	0.68 (0.50, 0.84)	0.68
LDL-C (mg/dL)	86 (75, 111)	93 (73, 116)	85 (76, 98)	89 (81, 115)	0.59
HDL-C (mg/dL)	60 (50, 68)	52 (47, 64)	61 (53, 69)	60 (47, 72)	0.13
TG (mg/dL)	114 (76, 175)	134 (88, 203)	106 (70, 168)	119 (62, 166)	0.32
C-reactive protein (mg/dL)	0.07 (0.03, 0.18)	0.09 (0.03, 0.25)	0.06 (0.02, 0.17)	0.06 (0.04, 0.14)	0.42
Medication					
DPP-4i	57 (69%)	13 (52%)	36 (82%)	8 (56%)	0.022*
SGLT2i	37 (45%)	15 (60%)	17 (39%)	5 (36%)	0.18
Biguanide	28 (34%)	14 (56%)	12 (27%)	2 (14%)	0.013*
GLP-1RA	17 (20%)	9 (36%)	4 (9%)	4 (29%)	0.021*
Statin	59 (71%)	15 (60%)	37 (84%)	7 (50%)	0.017*
Fibrate	16 (19%)	6 (24%)	8 (18%)	2 (14%)	0.73
Ezetimib	16 (19%)	3 (12%)	10 (23%)	3 (21%)	0.54
ACEi/ARB	42 (51%)	12 (48%)	22 (50%)	8 (57%)	0.85
MRA	10 (12%)	3 (12%)	6 (14%)	1 (7%)	0.81

The median age of all patients was 70 (61, 76) years, with 54% being male. The median body mass index was 23.8 (21.7, 25.2) (kg/m2). The median FIB-4 and APRI levels were 1.68 (1.18, 2.40) and 0.29 (0.23, 0.39), respectively. The median platelet, aspartate aminotransferase (AST), alanine transaminase (ALT), and γ-GTP were 20.2 (16.8, 23.9) ×10 4/μL, 22 (18, 27) IU/L, 21 (16, 30) IU/L, and 28 (19, 48) IU/L, respectively. The median initial dose of imeglimin was 2000 (1000, 2000) mg/day. The administration rates of dipeptidyl peptidase-4 inhibitor (DPP-4i), sodium-glucose cotransporter-2 inhibitor (SGLT2i), glucagon-like peptide-1 receptor agonist (GLP-1RA), and angiotensin converting enzyme inhibitor (ACEi)/angiotensin II receptor blocker (ARB) were 69%, 45%, 20%, and 51%, respectively. Significant differences across the three subgroups were observed in APRI, age, body mass index, hemoglobin, platelet, estimated glomerular filtration rate (eGFR), AST, γ-GTP, and the administration rate of DPP-4i, biguanide, GLP-1RA and statin.

Outcomes

Changes in Variables Three Months After the Initiation of Imeglimin Across Three Subgroups

**Figure 1:**
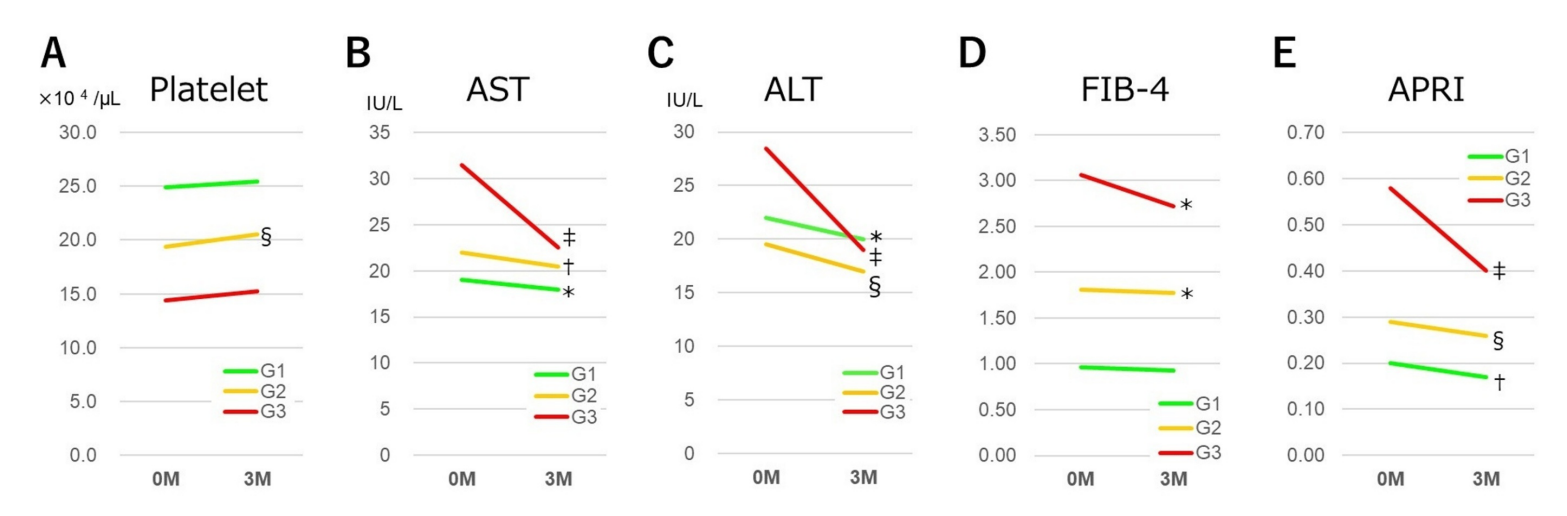
Changes in variables three months after the initiation of imeglimin across three subgroups Changes in platelet (A), AST (B), ALT (C), FIB-4 (D), and APRI (E) are presented. Significant differences are indicated as follows: * p < 0.05, † p < 0.01, ‡ p < 0.005, § p < 0.001. Abbreviations: ALT, alanine transaminase; APRI, aspartate aminotransferase to platelet ratio index; AST, aspartate aminotransferase; FIB-4 index, fibrosis-4 index.

Table 2 and Figure 1 present the changes in variables three months after the initiation of imeglimin.

**Table 2:** Changes in variables three months after the initiation of imeglimin across three subgroups Variables are presented as median (interquartile range). Statistical significance was assessed using the Wilcoxon signed-rank test. Asterisks (*) indicate statistically significant p-values. Abbreviations: ALT, alanine transaminase; APRI, aspartate aminotransferase to platelet ratio index; AST, aspartate aminotransferase; FIB-4 index, fibrosis-4 index; γ-GTP, γ-glutamyl transpeptidase; HbA1c, hemoglobin A1c

Variables	Overall (n = 83)			FIB-4 index								
				< 1.30 (G1, n = 25)			1.30 ≤, < 2.67 (G2, n = 44)			2.67 ≤ (G3, n = 14)		
	0M	3M	p-value	0M	3M	p-value	0M	3M	p-value	0M	3M	p-value
HbA1c (mg/dL)	8.4 (7.8, 9.8)	7.8 (7.2, 9.0)	< 0.001*	8.7 (7.8, 10.7)	8.0 (7.3, 9.6)	0.06	8.5 (7.9, 9.4)	8.0 (7.3, 8.7)	< 0.001*	8.1 (7.7, 9.6)	7.4 (6.8, 7.7)	0.005*
Platelet (×10^ 4^ /μL)	20.2 (16.8, 23.9)	20.8 (17.7, 25.1)	< 0.001*	24.9 (21.7, 26.9)	25.4 (24.1, 27.8)	0.19	19.4 (17.2, 21.5)	20.5 (18.3, 23.0)	< 0.001*	14.4 (12.8, 16.3)	15.3 (11.6, 17.6)	0.21
AST (IU/L)	22 (18, 27)	20 (16, 24)	< 0.001*	19 (16, 23)	18 (16, 21)	0.024*	22 (19, 26)	21 (17, 25)	0.005*	32 (26, 50)	23 (20, 28)	0.005*
ALT (IU/L)	21 (16, 30)	19 (14, 23)	< 0.001*	22 (18, 30)	20 (17, 23)	0.015*	20 (15, 26)	17 (13, 22)	< 0.001*	29 (18, 34)	19 (14, 23)	0.004*
γ-GTP (IU/L)	28 (19, 48)	26 (17, 39)	< 0.001*	31 (21, 46)	27 (17, 33)	0.011*	25 (18, 39)	20 (14, 38)	0.033*	53 (26, 120)	37 (25, 54)	0.040*
FIB-4 index	1.68 (1.16, 2.40)	1.68 (1.04, 2.22)	0.002*	0.96 (0.90, 1.12)	0.93 (0.76, 1.08)	0.69	1.81 (1.63, 2.18)	1.77 (1.58, 2.07)	0.046*	3.06 (2.81, 3.39)	2.72 (2.62, 3.17)	0.017*
APRI	0.29 (0.23, 0.39)	0.25 (0.19, 0.33)	< 0.001*	0.20 (0.16, 0.23)	0.17 (0.14, 0.21)	0.007*	0.29 (0.25, 0.37)	0.26 (0.22, 0.32)	< 0.001*	0.58 (0.42, 0.67)	0.41 (0.34, 0.46)	0.002*

HbA1c, AST, ALT, γ-GTP, FIB-4, and APRI demonstrated significant reductions overall, whereas platelet levels showed a significant increase. AST, ALT, and γ-GTP demonstrated significant decreases across all subgroups, whereas HbA1c showed significant reductions in G2 and G3. Platelet levels significantly increased in G2. FIB-4 displayed significant reductions in G2 (p = 0.046) and G3 (p = 0.017), while APRI was significantly reduced across all three subgroups (p = 0.007 in G1, p < 0.001 in G2, and p = 0.002 in G3).

Comparison of Changes in FIB-4 and APRI Across Three Subgroups

**Figure 2:**
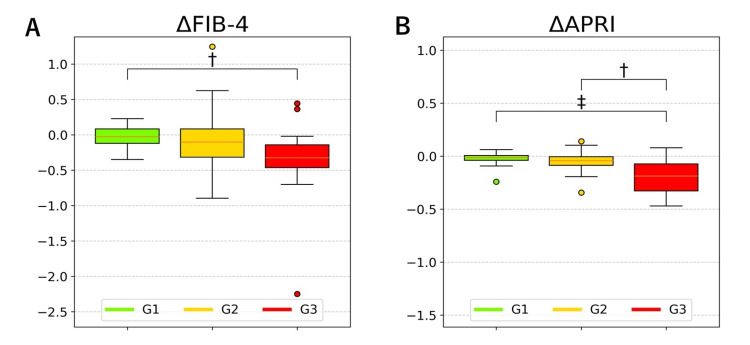
Comparison of changes (Δ) in FIB-4 and APRI across three subgroups ΔFIB-4 (A) and ΔAPRI (B) across three subgroups are presented. The central line in each box represents the median, while the box extends from the first to the third quartile. Whiskers indicate the minimum and maximum values within 1.5 times the interquartile range. Outliers are represented by circles. To improve visualization, extreme values were truncated. Significant differences are indicated as follows: p < 0.01 (†), p < 0.005 (‡). Abbreviations: APRI, aspartate aminotransferase to platelet ratio index; FIB-4 index, fibrosis-4 index.

Table 3 and Figure 2 provide a comparison of changes in FIB-4 and APRI across three subgroups.

**Table 3:** Comparison of changes (Δ) in FIB-4 and APRI across three subgroups Variables are presented as median (interquartile range). Statistical significance was assessed using the Kruskal-Wallis test. Asterisks (*) indicate statistically significant p-values. Abbreviations: APRI, aspartate aminotransferase to platelet ratio index; FIB-4 index, fibrosis-4 index

Variables	Overall (n = 83)	FIB-4 index			p-value
		< 1.30 (G1, n = 25)	1.30 ≤, < 2.67 (G2, n = 44)	2.67 ≤ (G3, n = 11)	
ΔFIB-4	-0.08 (-0.26, 0.07)	-0.03 (-0.12, 0.08)	-0.10 (-0.32, 0.08)	-0.33 (-0.46, -0.14)	0.012*
ΔAPRI	-0.04 (-0.09, -0.01)	-0.01 (-0.04, 0.01)	-0.04 (-0.09, -0.01)	-0.19 (-0.33, -0.07)	0.001*

Intergroup comparisons revealed significant differences in ΔFIB-4 and ΔAPRI (p = 0.012 for ΔFIB-4 and p = 0.001 for ΔAPRI). The ΔFIB-4 in G3 was significantly greater than that in G1 (p = 0.01), while the ΔAPRI in G3 was significantly greater than those in G1 (p = 0.004) and G2 (p = 0.007).

Baseline Parameters and Changes in Parameters Correlated With ΔFIB-4

Table 4 presents the baseline parameters correlated with ΔFIB-4 three months following the initiation of imeglimin.

**Table 4:** Baseline parameters correlated with ΔFIB-4: results of multiple regression analysis Standardized regression coefficients (β), 95% confidence intervals (CI), p-values, and variance inflation factors (VIF) are presented. Asterisks (*) indicate statistically significant p-values. Abbreviations: FIB-4 index, fibrosis-4 index; HbA1c, hemoglobin A1c; TG, triglyceride; γ-GTP, γ-glutamyl transpeptidase

Variables	β	95% CI	p-value	VIF
FIB-4 index	-0.45	(-0.72, -0.17)	0.002*	6.77
HbA1c	-0.13	(-0.32, 0.07)	0.20	4.55
γ-GTP	-0.09	(-0.37, 0.20)	0.54	3.11
TG	-0.27	(-0.49, -0.06)	0.011*	1.82

ΔFIB-4 was negatively correlated with baseline FIB-4 (β = -0.45, p = 0.002) and triglycerides (β = -0.27, p = 0.011). Table 5 presents the changes in parameters correlated with ΔFIB-4 three months after the initiation of imeglimin.

**Table 5:** Changes (Δ) in parameters correlated with ΔFIB-4: results of multiple regression analysis Standardized regression coefficients (β), 95% confidence intervals (CI), p-values, and variance inflation factors (VIF) are presented. Asterisks (*) indicate statistically significant p-values. Abbreviations: HbA1c, hemoglobin A1c; LDL, low-density lipoprotein; TG, triglyceride; γ-GTP, γ-glutamyl transpeptidase.

Variables	β	95% CI	p-value	VIF
Δγ-GTP	0.78	(0.58, 0.98)	0.002*	1.61
ΔHbA1c	-0.13	(-0.32, 0.06)	0.18	1.40
ΔTG	0.02	(-0.16, 0.20)	0.80	1.18
ΔLDL-C	-0.02	(-0.19, -0.15)	0.79	1.07

ΔFIB-4 showed a significant positive correlation with Δγ-GTP (β = 0.78, p = 0.002), whereas no significant correlation was observed with ΔHbA1c (β = -0.13, p = 0.18). It is essential to note that the causal relationship between these variables remains unclear.

## Discussion

Main findings

In patients with type 2 diabetes, liver enzyme levels, FIB-4, and APRI exhibited significant reductions from baseline, whereas platelet levels increased 12 weeks following imeglimin administration. The extent of reduction in FIB-4 and APRI following imeglimin administration was more pronounced in individuals with higher baseline FIB-4 levels. The reduction in FIB-4 was significantly correlated with baseline TG and the decrease in γ-GTP, whereas no significant correlation was observed with the change in HbA1c.

Previous studies

In a retrospective observational study of patients with MASLD and type 2 diabetes, AST and γ-GTP levels were significantly reduced 16 weeks after imeglimin administration, whereas ALT and FIB-4 levels remained unchanged [17]. These findings differ from those of the present study, possibly due to variations in the study populations. By 24 weeks, significant reductions were observed in AST, γ-GTP, ALT, and FIB-4 levels [17]. These results align with the findings of the present study. In the present study, significant reductions in AST, ALT, and γ-GTP, as well as FIB-4 and APRI, were observed 12 weeks after imeglimin administration, suggesting that its effects on liver enzymes may manifest relatively early. Pioglitazone [20], semaglutide, a glucagon-like peptide-1 receptor agonist (GLP-1RA) [21], tirzepatide, a dual glucose-dependent insulinotropic peptide and glucagon-like peptide-1 receptor agonist (GIP/GLP-1RA) [22], and tofogliflozin, a sodium-glucose cotransporter 2 inhibitor (SGLT2i) [23], have all been shown to improve liver function in participants with liver dysfunctions, including NAFLD, NASH (MASH), and hepatic fibrosis, as demonstrated in randomized controlled trials (RCTs). Reductions in liver enzymes were observed as early as 12 weeks after administration of these agents, and longer-term follow-up revealed histological improvements in the liver. In the present study, liver enzyme reductions were observed 12 weeks after imeglimin administration, aligning with the timing of enzyme reductions seen with the aforementioned antidiabetic agents that have demonstrated hepatoprotective effects. In contrast, the effectiveness of metformin, which shares a similar chemical structure with imeglimin, in ameliorating liver enzyme levels and histological abnormalities in NAFLD/NASH remains inconclusive [24].

Differential effects of metformin and imeglimin on hepatic mitochondrial respiratory chain complexes

Metformin targets mitochondrial respiratory chain complex I, diminishing the efficiency of mitochondrial respiration, thereby reducing adenosine triphosphate (ATP) production and suppressing gluconeogenesis [25]. Similar to metformin, imeglimin also reduced ATP production in cultured hepatocytes; however, its inhibitory effect on mitochondrial respiration was less pronounced than that of metformin. Imeglimin also activated adenosine monophosphate-activated protein kinase (AMPK), albeit to a lesser extent than metformin [26]. Imeglimin enhances fatty acid oxidation by restoring mitochondrial respiratory chain complex III activity, thereby mitigating hepatic steatosis. Additionally, imeglimin reduced reactive oxygen species accumulation and increased mitochondrial biogenesis. Through enhanced mitochondrial biogenesis, imeglimin inhibits hepatocyte apoptosis, attenuates macrophage infiltration, downregulates proinflammatory cytokine expression, and suppresses liver fibrosis progression [27]. Imeglimin, but not metformin, upregulated the expression of genes encoding proteins associated with mitochondrial respiratory complexes I and III [26]. These distinctions in the effects of imeglimin and metformin on hepatic mitochondrial respiratory chain complexes, as reported in previous studies, suggest that the hepatoprotective mechanisms of imeglimin may differ from those of metformin. This hypothesis-generating perspective is based on preclinical evidence and warrants further investigation in future mechanistic studies. Furthermore, it is estimated that imeglimin exerts a direct effect on the liver as mentioned above, which is not inconsistent with the lack of a significant correlation between the changes in HbA1c and the changes in FIB-4 index in this study.

Selection of medications for type 2 diabetes treatment

Type 2 diabetes requires a tailored preventive and therapeutic approach based on the pathophysiology of the disease. In Japan, multifactorial interventions, including blood glucose control, have been recognized as crucial for preventing complications. It has been recommended to select the appropriate medication from all classes of blood glucose-lowering drugs, taking into account the individual patient's disease state. This remains the case today, but the consensus statement issued in 2022 divided recommendations based on obesity (BMI ≥25 kg/m²) and non-obesity [28]. Although imeglimin was included for both categories, its priority was not set high due to limited data. On the other hand, in Western countries, metformin has long been recommended as the first-line treatment for managing type 2 diabetes due to its significant HbA1c-lowering effect, low risk of hypoglycemia, and low cost [29]. However, in 2022, the American Diabetes Association (ADA) updated the Standards of Medical Care in Diabetes, stating that treatment options should be considered based on each patient's pathophysiology, irrespective of baseline HbA1c levels, target HbA1c, or the use of metformin, particularly in patients with atherosclerotic cardiovascular disease (ASCVD) or its high risk, heart failure, or chronic kidney disease [30]. The findings of this study suggest that the degree of hepatic fibrosis may be a potential indicator when selecting treatment, but due to the design limitations of an observational study, further research is needed to draw clearer conclusions.

Clinical implication

Evidence suggests that imeglimin, alongside SGLT2 inhibitors, GLP-1 receptor agonists, dual GIP/GLP-1 receptor agonists, and thiazolidinediones, may be effective in ameliorating MASLD associated with type 2 diabetes. While the statistically significant reductions in FIB-4 and APRI observed in this study are promising, their clinical significance remains unclear, especially in the absence of histological or imaging confirmation. These findings should be interpreted with caution, and future studies are needed to determine whether such short-term changes in non-invasive fibrosis markers translate into meaningful long-term clinical outcomes. Furthermore, although metformin, which shares a similar chemical structure with imeglimin, exhibits inconsistent effects on MASLD, imeglimin may exert hepatoprotective effects through distinct mechanisms involving hepatic mitochondria. While this hypothesis is supported by previous experimental data, it remains speculative and was not directly evaluated in the present study.

Limitation

This study has several limitations. One limitation is the reliance on blood test results without imaging or pathological validation. Additionally, the sample size is relatively small, limiting the statistical power of the study. As a single-center, retrospective study, caution is required when generalizing the results. The observation period was short; however, metabolic diseases, including type 2 diabetes, are chronic conditions, and longer-term studies are necessary to evaluate the long-term efficacy and sustainability of interventions. Due to the short observation period, potential cardiovascular events, which may be associated with MASH, were not assessed and should be considered in future investigations. The results of this study suggest that the degree of liver fibrosis may serve as an indicator when selecting treatment medications. However, due to the design limitations of an observational study, including the potential for selection bias and lack of randomization, caution is warranted in interpreting causal relationships. Further research, ideally with prospective and controlled designs, is needed to draw more definitive conclusions.

## Conclusions

This study demonstrated that imeglimin administration in patients with type 2 diabetes led to significant reductions in liver enzyme levels, FIB-4, and APRI, with a concomitant increase in platelet levels after 12 weeks. Notably, greater reductions in FIB-4 and APRI were observed in individuals with higher baseline FIB-4 levels. The reduction in FIB-4 was significantly correlated with baseline triglyceride levels and the decrease in γ-GTP, but not with changes in HbA1c, suggesting a direct hepatic effect of imeglimin independent of glycemic control. The observed early reduction in liver enzyme levels aligns with findings reported for other antidiabetic agents with known hepatoprotective properties, including GLP-1 receptor agonists, dual GIP/GLP-1 receptor agonists, SGLT2 inhibitors, and thiazolidinediones. In conclusion, while imeglimin appears to be a promising therapeutic agent for MASLD in patients with type 2 diabetes, particularly those with higher baseline liver fibrosis indices, its long-term efficacy and clinical positioning require further investigation.
